# OT-Mation: an open-source code for parsing CSV files into Python scripts for control of OT-2 liquid-handling robotics

**DOI:** 10.1093/synbio/ysaf009

**Published:** 2025-04-11

**Authors:** Alex Laverick, Katherine Convey, Catherine Harrison, Jenny Tomlinson, Jem Stach, Thomas P Howard

**Affiliations:** School of Natural and Environmental Sciences, Newcastle University, Newcastle upon Tyne, NE1 7RU, United Kingdom; School of Natural and Environmental Sciences, Newcastle University, Newcastle upon Tyne, NE1 7RU, United Kingdom; School of Natural and Environmental Sciences, Newcastle University, Newcastle upon Tyne, NE1 7RU, United Kingdom; Plant Protection, Fera Science Ltd, York Biotech Campus, York YO41 1LZ, United Kingdoms; Plant Protection, Fera Science Ltd, York Biotech Campus, York YO41 1LZ, United Kingdoms; School of Natural and Environmental Sciences, Newcastle University, Newcastle upon Tyne, NE1 7RU, United Kingdom; School of Natural and Environmental Sciences, Newcastle University, Newcastle upon Tyne, NE1 7RU, United Kingdom

**Keywords:** Opentrons OT-2, robotic liquid handling, statistical design of experiments

## Abstract

OT-Mation is an open-source Python script designed to automate the programming of OT-2 liquid-handling robots, making combinatorial experiments more accessible to researchers. By parsing user-defined CSV files containing information on labware, reagents, pipettes, and experimental design, OT-Mation generates a bespoke Python script compatible with the OT-2 system. OT-Mation enhances reproducibility, reduces human error, and streamlines workflows, making it a valuable addition to any laboratory utilizing OT-2 robotics for liquid handling. While OT-Mation can be used for setting up any type of experiment on the OT-2, its real utility lies in making the connection between multifactorial experimental design software outputs (i.e. design of experiments arrays) and liquid-handling robot executable code. As such, OT-Mation helps bridge the gap between code-based flexibility and user-friendly operation, allowing researchers with limited programming skills to design and execute complex experiments efficiently.

## Graphical Abstract

**Figure:**
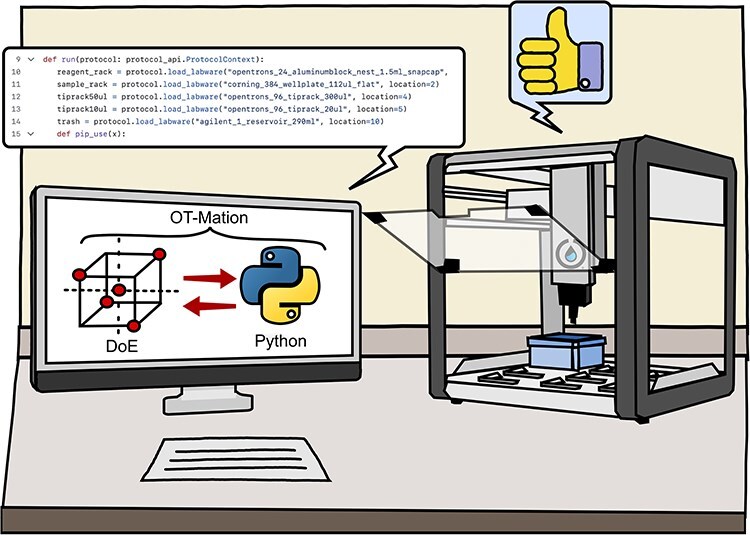

## 1. Introduction

Automation of small-scale liquid handling is gaining traction in life sciences, allowing researchers to conduct high throughput or combinatorial experiments with reduced human error, greater accuracy, and traceable workflows [1]. A combination of low cost and flexibility makes their incorporation into existing workflows an attractive option for many researchers [2–4]. There is also recognition that a large proportion of scientific research cannot be easily replicated and that part of the problem may be the quality and consistency of protocol reporting [5–7]. The explicit and shareable protocols used by liquid-handling robotics may therefore be a useful tool to improve data quality and reproducibility [1]. Another contributing factor may be the use of the one-factor-at-a-time (OFAT) experimental approach. This persists despite well-documented drawbacks, including (i) intuition, narrative, and confirmation biases and (ii) sensitivity to unknown confounding factors [8, 9]. Automated liquid handling can increase the combinations of experiments that can be handled by an individual researcher, facilitating better-designed experiments with more robust outcomes.

Combining automated liquid-handling robotics with statistical design of experiments (DoE) offers a powerful, iterative approach to understanding and optimizing complex biological systems. DoE provides a robust framework for experimental design, minimizing experimental effort whilst maximizing knowledge gained. Compared to the traditional OFAT approach, DoE separates real effects from experimental noise with far greater efficiency and uncovers multifactorial interactions [8, 9]. When coupled with modern approaches to data analysis, it provides predictive models for experimental optimization. Synthetic biology already applies DoE in a range of settings [10], including understanding and manipulating metabolic pathways [11–13], engineering biosensors [14], and optimizing cell-free systems [15–17].

Despite the apparent synergy between DoE and automated liquid handling, the ability to couple these two approaches is not straightforward. Protocol development for liquid-handling robotics is typically via either a graphical user interface (GUI) or a computer programming language. Creating a simple protocol through a GUI is quick and easy and offers an excellent entry into the use of robotics for new users. Implementing and editing more complex procedures is difficult and may take more time than running the protocol [15, 18]. By contrast, some liquid-handling platforms permit direct programming of the robotics, allowing users to develop more bespoke protocols. The OT-2 system (opentrons.com) operates through Python scripts providing flexibility and control. In addition, tools such as LabOP [19] and PyLabRobot [20] provide cross-platform operability and protocol flexibility. In each case, however, these methods require skills that many laboratory-focused researchers are not confident in [21], coupled with the need to learn the relevant nomenclature and structure. Furthermore, while multifactorial experiments can be set up using code, critically DoE is a sequential optimization process that frequently requires fold-over or augmentation of experimental designs with new combinations of factor settings [9], necessitating a different script for each sequential experiment. Finally, both methods require human intervention to transfer experimental arrays into the liquid-handling protocols, which may also introduce errors in the process. Neither GUI nor direct programming is therefore easily adapted for the multifactorial experiments required by DoE.

Here, we describe OT-Mation, a Python script that automates and simplifies the programming steps of the OT-2 without sacrificing the utility of code-based development. OT-Mation generates a bespoke Python script for control of the OT-2 from four user-defined comma-separated value (CSV) format files. These files provide information on the labware on the deck of the robot, the reagents required for the experiment, pipetting hardware, and the experimental design parameters. In practice, only the Experimental Parameters file needs modification between experiments, and its format aligns with experimental arrays from statistical design software, eliminating the need for manual data entry into a GUI or Python code.

## 2. Materials and methods

OT-Mation was developed in Python 3.7 and 3.9 and is currently in use in Python 3.12 on both Windows and MacOS. Testing of the script was performed on an Opentrons OT-2 liquid-handling platform.

## 3. Results and discussion

### 3.1 Design considerations

OT-Mation was designed with flexibility and iteration in mind. The script is capable of supporting complex, iterative workflows—such as DoE optimizations in which different multifactorial experimental arrays are required at each iteration. A further consideration for iterative, multifactorial protocols is the ability for dilutions to be calculated as part of the script-generating process. This is especially useful where there are several different concentrations of a reagent required across the experiment, but they must be generated from one single stock solution. We therefore designed OT-Mation such that it calculates how the robot should dilute from a stock concentration to a target concentration and that the robotic steps to achieve this are included in the output script. Without this capability, all dilution calculations would be done in advance by the researcher prior to manual inclusion in their Python script. Finally, OT-Mation uses a broad variety of commands enabling users to specify settings for each reagent giving the user control and flexibility over their experiments. While our primary motivation was to support sequential multifactorial experimentation as required when using DoE, the versatility of OT-Mation makes it suitable for a variety of experimental procedures (e.g. serial dilutions or setting up PCR). In a statistically designed experimental array, there are multiple experimental runs in which the concentrations (or sometimes a categorical selection) of multiple factors are adjusted simultaneously and systematically. When run, OT-Mation generates an output script that once written, remains the same. This allows scripts to be archived alongside experimental data, acting as a record of experiments carried out.

### 3.2 File structure

**Figure 1.**
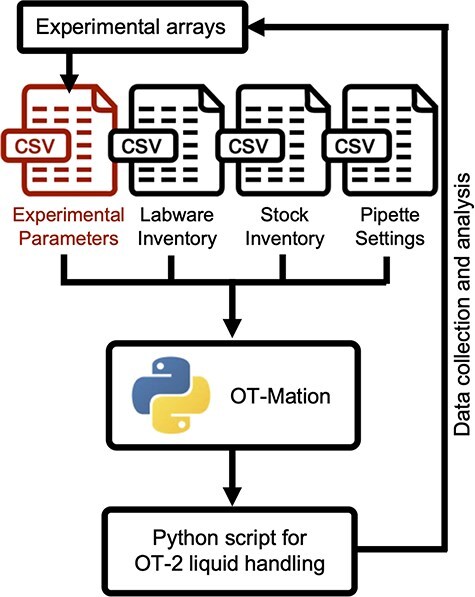
OT-Mation workflow. OT-Mation was designed to support the iterative, sequential workflow required in statistical DoE but can be applied in any iterative design–build–test–learn cycle. In a cyclic experimental process, after results from an experiment have been analysed, new factor combinations or parameters are chosen. Using OT-Mation, only the ‘Experimental Parameters’ CSV file (red) needs to be changed to generate a new protocol—and this is compatible with the output format of many statistical packages removing the need for human intervention in transferring data.

**Figure 2.**
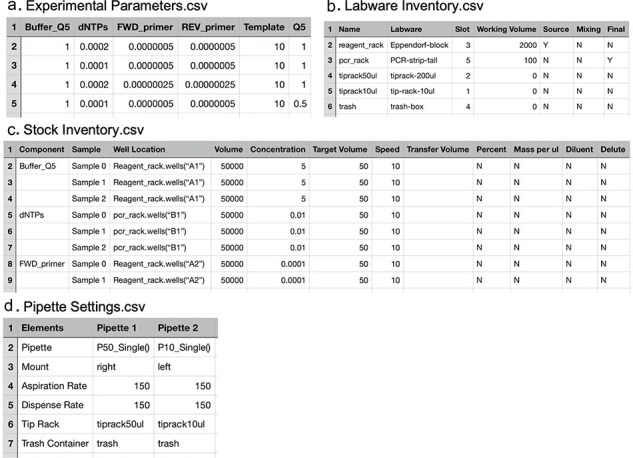
Layout of the four user defined CSV Files central to OT-Mation. A. Experimental Parameters. B. Labware Inventory. C. Stock Inventory. D. Pipette Settings.

OT-Mation parses the data contained within four CSV files to generate an output Python script that is uploaded onto the OT-2 through the Opentrons companion application (Fig. 1). The four CSV files are ‘Experimental Parameters’, ‘Labware Inventory’, ‘Stock Inventory’, and ‘Pipette Settings’ (Fig. 2). OT-Mation calculates the pipetting actions for the robot to complete the experiment without requiring the user to write any code.

#### 3.2.1 Experimental Parameters file

The Experimental Parameters file is the primary route by which experimental designs are parsed into OT-Mation. Between experimental iterations, this is the only file that needs to be altered. Each row represents a sample being assembled and each column a reagent (Fig. 2a). Each row represents an experimental run as identified in the experimental array: the first row is for the headers, the second row is Sample 0, the third row is Sample 1, and so forth. In each cell, the desired final concentration of the reagent in the sample is entered. If 0 is entered, the reagent is skipped. OT-Mation calculates the volume of Diluent required to reach the target volume of the sample. If the number of rows within the Stock Inventory exceeds the rows of Experimental Parameters, OT-Mation will produce an error.

#### 3.2.2 Labware Inventory file

All labware to be used in the OT-2 is specified in the Labware Inventory CSV file (Fig. 2b). Each row specifies one piece of labware, and all information about that labware is found within seven columns: ‘Name’, ‘Labware’, ‘Slot’, ‘Working Volume’, ‘Source’, ‘Mixing’, and ‘Final’. The ‘Name’ column identifies the name used within the Python script, while the ‘Labware’ column specifies the labware according to the Opentrons labware library. ‘Slot’ refers to which working position the labware will be in, as the OT-2 has 11 fixed labware slots. ‘Working Volume’ specifies the maximum volume each piece of labware can hold, given in microlitres. Labware that is not used for mixing and storing samples, such as tip racks and trash, can be assigned values of 0. A piece of labware is designated as ‘Source’, ‘Mixing’, or ‘Final’ by marking the cells in the last three columns with a Y (yes) or N (no). Labware designated as a ‘Source’ will only be used as a location to aspirate reagents from, ‘Mixing’ identifies if the labware is acting as an intermediary mixing platform, and ‘Final’ identifies labware as the final destination for samples. The information in ‘Source’, ‘Mixing’, and ‘Final’ is combined with data from the Experimental Parameters CSV file to write the protocol and determine how reagents are handled.

#### 3.2.3 Stock Inventory file

The stock inventory is recorded across columns with each reagent represented across multiple rows according to the concentration of the reagent in each sample (Fig. 2c). Entries in the ‘Component’ column are parsed by OT-Mation and designated as keys for the dictionary used to store experimental sample information. If a cell is blank, the components designation is considered the same as the previously named entry. ‘Sample’ refers to the assembly of each sample by the OT-2. This starts at 0 as this is the initial value for lists and counts in Python. ‘Well Location’ refers to where the reagent stock is stored on the OT-2 work surface and what piece of labware it is stored in. ‘Volume’ indicates the volume of the stock available, in microlitres. ‘Concentration’ indicates the concentration of the stock solution. The concentration can be recorded as percentage, mass per microlitre, or mols. ‘Target Volume’ refers to the final volume of the sample in microlitres. Information recorded in the ‘Speed’ column sets a default value for both aspirating and dispensing rates in microlitres per second. Values entered here overwrite the default speed in Pipette Settings. ‘Transfer Volume’ is the specific amount of a reagent to be dispensed into the samples. If cells in this column are left blank, OT-Mation will calculate which transfers need to occur to create a sample containing the correct concentration of reagents. Users can specify if a reagent is for diluting a sample or another reagent, or if the reagent needs to be diluted by a diluent before it is used in sample assembly by entering Y or N in the ‘Dilutant’ and ‘Diluent’ columns. Reagents are handled by the OT-2 in the order they are listed in the Stock Inventory file, distributing reagent A into all samples before reagent B is used.

#### 3.2.4 Pipette Settings file

The Pipette Settings CSV contains the settings for the pipettes used throughout the assembly of the samples (Fig. 2d). Two pipettes can be mounted simultaneously in the OT-2. The columns of the Pipette Setting CSV file represent the two pipettes, and each row specifies their settings, including ‘Pipette’, ‘Mount’, ‘Aspirate Rate’, ‘Dispense Rate’, ‘Tip Rack’, and ‘Trash Container’. ‘Pipette’ specifies which model of pipette (P10, P50, etc.) is being used. This needs to be entered as it appears in the Opentrons API. ‘Mount’ indicates which mount the pipette is affixed to, either left or right. ‘Aspirate Rate’ and ‘Dispense Rate’ set the aspiration rate and dispense rate of the pipettes, respectively, in microlitres per second. If a speed is not specified in the Stock Inventory CSV for a specific reagent, the aspirate and dispense rates in Pipette Settings are used. ‘Tip Rack’ allocates a tip rack for the corresponding pipette. The names entered in these cells must match names given to tip racks in the Labware Inventory. ‘Trash Container’ assigns a location for each pipette to eject tips. OT-Mation automatically calculates which pipette to use based on the volume that needs to be transferred at each step.

### 3.3 Implementation

OT-Mation runs in the same way as a standard Python script, through the user’s preferred integrated development environment or text editor. The four CSV files must be within the same working directory as the OT-Mation script to be read. Alternatively, OT-Mation can be edited to contain file roots to the CSV files of interest. Within the OT-Mation script, there are spaces designated for the file names of the four CSV files, and for the file name of the output script (Supplementary Figure S1). This is the only time users need to edit the Python script. As the CSV files reside in the same folder as OT-Mation, OT-Mation can be copied into multiple folders with the files of interest to keep work separate [22].

OT-Mation reads the file contents in a specific order: Stock Inventory, Experimental Parameters, Pipette Settings, and finally Labware Inventory, with Stock Inventory serving as a reference for dictionary assembly. Dictionaries ascribe ‘Keys’ to ‘Values’, for rapid access to CSV data, which is then stored in nested dictionaries (Supplementary Figure S2). Nested dictionaries efficiently catalogue data for automated scripts, enabling fast calculations for dilutions, well transfers, reagent stocks, and target concentrations without affecting unrelated data.

After running OT-Mation, a Python output script is generated in the same working directory as the OT-Mation script. Every time the OT-Mation script is run, a new output script will overwrite the existing output script. The output script can be uploaded to the Opentrons companion application, which reads the script and generates instructions for the robot to carry out the experiment. Copies of the output script can be created and shared with other researchers allowing users to share and reproduce experiments. Finally, though not implemented in its current version, the open-source nature of OT-Mation allows users to modify the output format of automation scripts. This means that the scripts can be modified to comply with third-party protocol sharing standards, for example, the LAP Repository and LAP Format [23].

## 4. Conclusion

OT-Mation improves the accessibility and efficiency of automated liquid handling for multifactorial experiments using the OT-2 robotic system. By enabling the generation of bespoke Python scripts from user-defined CSV files, OT-Mation bridges the gap between the flexibility of code-based robotics control and the user-friendliness required for broader adoption in laboratory settings. Typically, only the Experimental Parameters file needs to be altered between experiments. As such, this tool simplifies the programming process and allows researchers with limited coding experience to leverage the full capabilities of the OT-2. As a result, OT-Mation both increases the accessibility of advanced liquid-handling automation and enhances the reproducibility and accuracy of complex multifactorial experiments.

## Supplementary Material

ysaf009_Supp

## Data Availability

Code, documentation, example files, and installation instructions can be found at https://github.com/tphoward/OT-Mation_Repo/.
