# Supplementary material for: OT-Mation: an open-source code for parsing CSV files into Python scripts for control of OT-2 liquid-handling robotics
Source: Synth Biol (Oxf). 2025 Apr 11;10(1):ysaf009. doi: 10.1093/synbio/ysaf009 (PMC12063526; doi:10.1093/synbio/ysaf009)
Supplement: ysaf009_Supp [file ysaf009_supp.zip › suppl_data/Laverick et al. OT-Mation Supplemental Figures.pdf]

```
Exp_parameters = "Experimental Parameters.csv"
Stock_inventory = "Stock Inventory.csv"
Pipette_settings = "Pipettes Settings.csv"
Labware_definitions = "Labware Inventory.csv"
Output_file = "Opentrons 36 GG.py"
```

Figure S1. File declarations. OT-Mation File declarations occur within the first five lines of the OT-Mation script meaning that users need only change the coloured text within the quotations marks of the declarations to match the CSV files they wish OT-Mation to read. Users can also specify the name of the output file which will be generated in the same working directory.

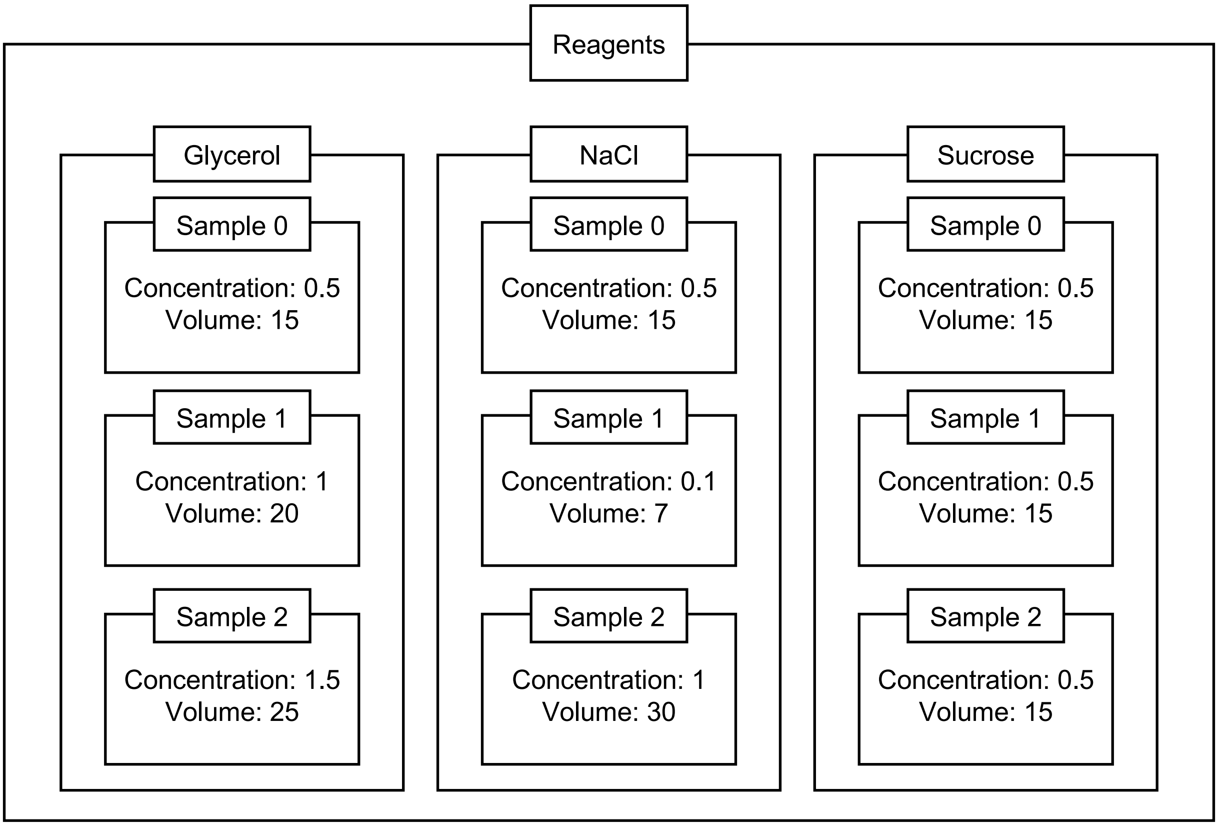

Figure S2. Graphical representation of dictionary data structure within OT- Mation. Values are saved to keys within a dictionary, with values being able to take on most data types available in Python. The Reagents dictionary contains each reagent as a key, the value saved to each reagent key is a nested dictionary where the keys are sample identifiers with values stored as another dictionary. This thrice nested dictionary contains keys matching attributes of interest for the reagent in a given sample such as Concentration as the key and '1' as the value.
